# Antimicrobial Air Filters Using Natural *Euscaphis japonica* Nanoparticles

**DOI:** 10.1371/journal.pone.0126481

**Published:** 2015-05-14

**Authors:** Gi Byoung Hwang, Ki Joon Heo, Ji Ho Yun, Jung Eun Lee, Hee Ju Lee, Chu Won Nho, Gwi- Nam Bae, Jae Hee Jung

**Affiliations:** 1 Center For Environment, Health, and Welfare Research, Department of Energy and Environmental Engineering, Korea University of Science and Technology (UST), Korea Institute of Science and Technology (KIST), Seongbuk-gu, Seoul, Republic of Korea; 2 Functional Food Center, Korea Institute of Science and Technology (KIST Gangneung Institute), Gangneung, Gangwon-do, Republic of Korea; 3 Aerosol and Bioengineering Laboratory, College of Engineering, Konkuk University, Hwayang-dong, Gwangjin-gu, Seoul, Republic of Korea; 4 Materials Chemistry Research Centre, Department of Chemistry, University College London, 20 Gordon Street, London, United Kingdom; 5 Han-River Environment Research Center, National Institute of Environmental Research (NIER), Yangseo-myeon, Yangpyeong-gun, Gyeonggi-do, Republic of Korea; Peking University, CHINA

## Abstract

Controlling bioaerosols has become more important with increasing participation in indoor activities. Treatments using natural-product nanomaterials are a promising technique because of their relatively low toxicity compared to inorganic nanomaterials such as silver nanoparticles or carbon nanotubes. In this study, antimicrobial filters were fabricated from natural *Euscaphis japonica* nanoparticles, which were produced by nebulizing *E*. *japonica* extract. The coated filters were assessed in terms of pressure drop, antimicrobial activity, filtration efficiency, major chemical components, and cytotoxicity. Pressure drop and antimicrobial activity increased as a function of nanoparticle deposition time (590, 855, and 1150 µg/cm2_filter_ at 3-, 6-, and 9-min depositions, respectively). In filter tests, the antimicrobial efficacy was greater against *Staphylococcus epidermidis* than *Micrococcus luteus*; ~61, ~73, and ~82% of *M*. *luteus* cells were inactivated on filters that had been coated for 3, 6, and 9 min, respectively, while the corresponding values were ~78, ~88, and ~94% with *S*. *epidermidis*. Although statistically significant differences in filtration performance were not observed between samples as a function of deposition time, the average filtration efficacy was slightly higher for *S*. *epidermidis* aerosols (~97%) than for *M*. *luteus* aerosols (~95%). High-performance liquid chromatography (HPLC) and electrospray ionization-tandem mass spectrometry (ESI/MS) analyses confirmed that the major chemical compounds in the *E*. *japonica* extract were 1(ß)-*O*-galloyl pedunculagin, quercetin-3-*O*-glucuronide, and kaempferol-3-*O*-glucoside. *In vitro* cytotoxicity and disk diffusion tests showed that *E*. *japonica* nanoparticles were less toxic and exhibited stronger antimicrobial activity toward some bacterial strains than a reference soluble nickel compound, which is classified as a human carcinogen. This study provides valuable information for the development of a bioaerosol control system that is environmental friendly and suitable for use in indoor environments.

## Introduction

Bioaerosols, which are aerosols of biological origin, may include intact microorganisms and/or parts or products of organisms [1]. Among them, airborne viruses, bacteria, and fungi have been investigated actively because airborne pathogens are readily transmitted by airflow and can cause a variety of diseases, including allergic rhinitis, asthma, chronic obstructive pulmonary disease (COPD), influenza, and severe acute respiratory syndrome (SARS) [2–5].

Over the last several decades, much effort has been devoted to develop efficient bioaerosol control methods and devices, including thermal methods [6–8], ultraviolet irradiation [9–11], antimicrobial filters [12,13], and titanium dioxide catalysis [14,15]. Among these, antimicrobial air filtration technologies are considered promising because they are easily applied to conventional air-conditioning systems. Previous studies have shown that air filtration technologies employing antimicrobial inorganic nanoparticles are effective in controlling bacterial aerosols. The antimicrobial efficacies of such systems depend on the exposure time, particle size, and concentration [16,17]. In particular, silver (Ag) nanoparticles are antimicrobial agents with a broad antimicrobial spectrum. Ag nanoparticles damage bacterial cell membranes and induce metabolic changes by decreasing enzyme activity [18,19]. Due to the outstanding antimicrobial activity of these materials, they have been extensively studied and applied in a variety of fields including indoor air quality (IAQ) and human health, air filtration, clothing manufacturing, electronics, food processing, cosmetics, and medical devices [20,21]. Similarly, copper (Cu) nanoparticles are widely known as antimicrobial substances. Previous studies showed that *Staphylococcus aureus*, *Escherichia coli*, *Bacillus subtilis*, *Klebsiella pneumoniae*, and *Pseudomonas aeruginosa* are sensitive to Cu nanoparticles [22]. Carbon nanotubes (CNTs) have also been applied to the control of water quality and IAQ. In their aquatic dispersion, CNTs showed strong antimicrobial activities as the reduction in bacterial viability reached a maximum of ~6 log, and in combination with Ag nanoparticles enhanced the antimicrobial activity of air filters. CNTs in direct contact with bacterial cells induce membrane damage and subsequent cell death. Single-walled CNTs are more toxic to bacteria than multi-walled CNTs [23–25].

Despite these advantages, inorganic nanoparticles [26,27] can exert adverse effects on health [28–30]. Previous studies have indicated that Ag nanoparticles are toxic to mammalian cells and certain organs because of transcutaneous penetration of the particles. Copper oxide nanoparticles induce DNA damage and oxidative stress in cells [31–34]. Various toxicity mechanisms for CNTs have been reported, including the interruption of transmembrane electron transfer, penetration of the cell envelope, and oxidation of cell components [35,36]. Moreover, long-term exposure or inhalation of these nanoparticles can lead to a reduction in respiratory functions [37,38].

To overcome these disadvantages, alternative air filtration technologies employing natural antimicrobial materials have been proposed [39–41]. Natural products, such as plant extracts, are typically less toxic relative to inorganic antimicrobial materials [42]. Natural-product nanoparticles consist of multiple compounds with various chemical properties. Depending on the extract and the nature of the material, these properties can include antibiotic activities such as anti-inflammatory, antiviral, and/or antimicrobial effects [43–46]. Many natural antimicrobial products have been discovered, including extracts from *Ratibida latipalearis*, *Teloxys graveolens*, *Dodonaea viscosa*, *Hyptis albida*, *Melaleuca alternifolia* (tea tree oil), and *Sophora flavescens* [47–50]. In addition, various chemical compounds contained in natural products have been shown to control bacterial metabolism. Recently, reports detailing the control of bacterial aerosols using natural-product nanoparticles have shown that extracted essential oils can reduce bacterial loads when applied to contaminated ventilation systems [51]. Air filters coated with tea tree oil inactivated ~99% of bacteria on their surface within 2–8 min. Filters coated with *S*. *flavescens* nanoparticles inactivated >91% of bacteria within 2 min [12,41,51].

In this study, a *Euscaphis japonica* extract was used to produce natural-product nanoparticles that were deposited onto air filters. Extracts of *E*. *japonica*, a tree grown in Northeastern Asia, are an ingredient in traditional herbal medicines used primarily to treat inflammation [52]. Several antibiotic activities of *E*. *japonica* have been reported, including anti-fibrotic [53], antiproliferative, and antimutagenic activities [54]. However, the antimicrobial activity of *E*. *japonica* when used in an air filtration system has not been reported. *E*. *japonica* nanoparticles were produced by a nebulization-thermal drying process [55,56]. The characteristics of *E*. *japonica* nanoparticle-coated filters were evaluated in terms of filtration efficiency, pressure drop, and antimicrobial activity under various particle deposition conditions. Additionally, the major components of the extract were analyzed using high-performance liquid chromatography-electrospray ionization tandem mass spectrometry (HPLC-ESI/MS/MS) and the cytotoxicity of *E*. *japonica* was compared with that of a soluble nickel compound (SNC), a known human carcinogen.

## Materials and Methods

### Preparation of *Euscaphis japonica* methanolic extract powder

*E*. *japonica* was purchased from a plant extract bank at the Korea Research Institute of Bioscience & Biotechnology (KRIBB), dried at room temperature, and pulverized in a blender. Pulverized *E*. *japonica* was extracted with methanol (106,009; Merck KGaA, Darmstadt, Germany) and sonicated for 3 days. Sonication was performed for 15 min, 10 times per day. The extracts were passed through nonfluorescent cotton filters (0.45 μm pore size, 13 mm filter diameter; Smartpor GHP syringe filter, Woongki Science, Seoul, Republic of Korea) and concentrated *in vacuo* to yield the methanol extract. After concentration, the extract was lyophilized for 24 h and the resulting *E*. *japonica* extract powder was stored at 5°C.

### Preparation of *E*. *japonica* nanoparticle-coated filters

**Fig 1:**
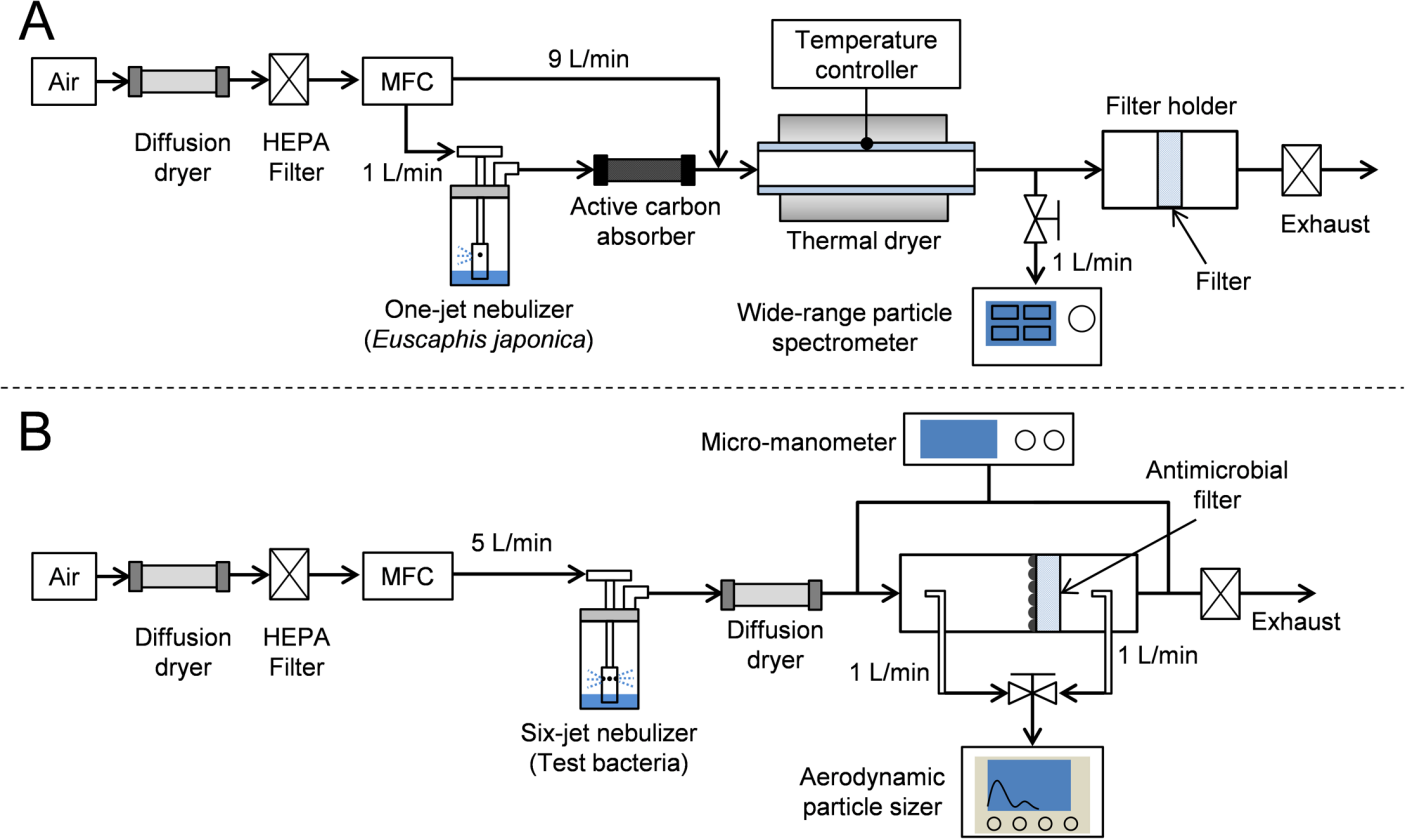
Experimental configurations of (a) the nebulization-thermal drying process used to fabricate *E*. *japonica* extract nanoparticle-coated filters, and (b) the antimicrobial filters, and pressure drop and filtration tests.

Antimicrobial filters were coated with *E*. *japonica* nanoparticles. Then 0.25 g of *E*. *japonica* extract powder was dissolved in 40 mL of methanol and filtered through a 0.45-μm cellulose acetate membrane filter (National Scientific Co., Rockwood, TN, USA) to remove insoluble residues. Fig 1 (A) shows the experimental setup for the fabrication of nanoparticle-coated filters. Twenty milliliters of the above *E*. *japonica* solution was poured into a one-jet Collison nebulizer (BGI Inc., Waltham, MA, USA). The nebulizer was supplied with 1 L/min of HEPA-filtered air under 1 psig. The resulting *E*. *japonica* aerosol was passed through an activated carbon absorber, mixed with 9 L/min of clean and dry air, and passed through a thermal glass quartz tube heater to remove methanol. The size and number concentration of the fabricated natural-product nanoparticles were measured using a wide-range particle spectrometer (WPS-100XP; MSP Co., Minneapolis, MN, USA) calibrated to a size range of 10–10,000 nm. The particle morphology was examined using a scanning electron microscope (200 NANO SEM; FEI Co., Hillsboro, OR, USA). The fabricated natural-product nanoparticles were continuously deposited onto polyurethane resin fiber filters (Clean & Science Co., Ltd., Seoul, Republic of Korea) (fiber diameter: 10–20 μm; thickness: 0.3 mm; and packing density: 33%).

### Test bacteria

*Staphylococcus epidermidis* (Korean Collection for Type Cultures KCTC 1917; Biological Resource Center, Republic of Korea) and *Micrococcus luteus* (KCTC 9856) were used as the test bacteria in this study. Gram-positive bacteria are widely used in bioaerosol research [57] and are common in indoor environments and on human skin [58,59]. The bacteria were incubated in a nutrient broth medium (Becton Dickinson, Franklin Lakes, NJ, USA) at 37°C. When the optical density of the bacterial suspension reached ~0.8 at 600 nm, the bacteria were harvested by centrifugation and washed three times with distilled water. The concentration of the resulting suspension was ~10^8^ colony forming units (CFU)/mL. One milliliter of the bacterial suspension was mixed with 19 mL of distilled water and loaded into a six-jet Collison nebulizer (BGI Inc.).

### Filtration efficiency and pressure drop

Fig 1 (B) shows a schematic diagram of the apparatus used to measure the filtration efficiency, pressure drop, and antimicrobial activity of the coated filters. Droplets containing test bacteria were sprayed using a six-jet Collison nebulizer supplied with a 5 L/min airflow under 1 psig. Moisture was removed from the droplets by passing through a diffusion dryer, and the bacterial aerosols were introduced onto the surface of the filter medium. The particle size and concentration of bacterial aerosols were measured with an aerodynamic particle sizer (APS model 3321; TSI, Inc., Shoreview, MN, USA) at both the inlet and outlet of the filter holder.

Filtration efficiencies of the coated filters were calculated using the following equation:

$$\eta \;=1-\frac{{\text{C}}_{\text{outlet}}}{{\text{C}}_{\text{inlet}}}\;,$$ (1)

where *C*_*inlet*_ and *C*_*outlet*_ represent the particle concentrations (particles/cm^3^_air_) of the bacterial aerosol at the inlet and outlet of the filter, respectively. The pressure drops of the coated filters were measured using a micromanometer (FC012; Furness Control, Ltd., Bexhill, UK).

### Antimicrobial tests

The bacterial aerosols were deposited onto the filters for ~3 min. After a contact time of 10 min, the filters were placed in 5 mL (*V*_*extraction*_) of phosphate-buffered saline (PBS) containing 0.01% Tween 80 and sonicated for 10 min to transfer the bacteria from the filters to the PBS solution. The resulting bacterial suspension was serially diluted onto plates of nutrient agar (Becton Dickinson) and incubated at 37°C for 24 h. The colonies that grew on the plates were counted. Bacterial inactivation efficiency was calculated as follows:

$$\text{ABCF}=\frac{CFU_{control}}{N_{control}},$$ (2)

$$\text{ABAF}=\frac{CFU_{antimicrobial}}{N_{antimicrobial}}\;,$$ (3)

$$N_{\text{control}}\;\text{or}\;N_{\text{antimicrobial}}=\frac{{\text{C}}_{\text{inlet}}\cdot {\text{Q}}_{\text{sampling}}\cdot \eta \cdot \zeta_{\text{extraction}}}{V_{\text{extraction}}}\;,$$ (4)

$$\text{Ratio of bacterial inactivation}\;=1-\;\frac{\text{ABAF}}{\text{ABCF}}\;,$$ (5)

where ABCF is the active proportion of bacteria from the control filter and ABAF is the active proportion of bacteria from the antimicrobial filter; *CFU*_*control*_ and *CFU*_*antimicrobial*_ are the concentrations (CFU/mL) of active bacterial suspensions produced from the control and antimicrobial filters, respectively; and *N* is the concentration of bacteria (particles/mL) in the extraction suspension that was plated onto agar. *Q*_*sampling*_ is the total airflow sampling volume and *ζ*_**extraction**_ is the physical extraction efficiency of the filter for bacteria, which is defined as the ratio of the number of particles transferred from the filter to the extraction liquid to the number of particles removed from the airflow using the filter. In this study, we assumed that the physical extraction efficiency for bacterial particles from all filters was identical.

### Chemical analysis of the *E*. *japonica* extract

Identification of the major chemical compounds in the *E*. *japonica* extract was performed using an HPLC-ESI/MS device (Thermo Fisher Scientific Inc., San Jose, CA) equipped with an ACCELA photodiode array detector (PDA), an autosampler, a quaternary pump, and an LCQ FLEET ion trap with an electrospray ionization source. The Thermo Xcalibur software (version 2.1) was used for data acquisition and processing. The mobile phase consisted of 0.1% formic acid in water (solvent A) and 0.1% formic acid in acetonitrile (solvent B). The gradient mode was as follows: 0–5 min, initial mobile phase solvent A/B (90:10, v/v); 5–40 min, linear gradient 30:70; 40–50 min, isocratic mode 30:70, and reconditioning steps to initial conditions for 15 min. A Waters Acquity BEH C18 column (3.0 × 100 mm, 1.7 m; Waters, Milford, MA, USA) was used for chemical separation of the *E*. *japonica* extract. The LC pump solvent flow rate was 150 mL/min. The sample injection volume was 5 μL. The ESI/MS conditions were as follows: positive and negative dual ion mode; mass range, *m/z* 100–1000; capillary voltage, 49 V; tube lens, 100 V; sheath gas flow rate (N_2_), 35 arb; auxiliary gas flow rate (N_2_), 10 arb; capillary temperature, 300°C.

### *In vitro* cytotoxicity of *E*. *japonica* nanoparticles

A549 human lung adenocarcinoma cancer cells and HEL 299 human lung fibroblasts were obtained from the American Type Culture Collection (ATCC, Rockville, MD, USA) and the cells were maintained in a 5% CO_2_ humidified atmosphere at 37°C. Eagle's minimum essential medium (EMEM; ATCC) was used to support the HEL 299 cells and RPMI-1640 (Hyclone, Logan, UT, USA) was used for A549 cultivation. These cells were supplemented with 10% (v/v) fetal bovine serum (FBS), 100 U/mL penicillin and 100 μg/mL streptomycin.

Cell viability upon exposure to *E*. *japonica* extract was evaluated using an EZ-Cytox cell viability assay kit (Daeil Lab Service, Ltd., Seoul, Republic of Korea) according to the manufacturer’s instructions. Cells (1 × 10^4^ cells/well) were plated in 96‑well plates, incubated at 37°C for 24 h, and given a fresh change of medium containing *E*. *japonica* extract at the indicated concentration for 48 h. At the end of the incubation, 10 μL of EZ-Cytox solution was added to the well and incubated for at least 1 more hour. The absorbance at 450 nm was measured using a Synergy HT Multi-microplate reader (BioTek Instruments, Winooski, VT, USA). Data were expressed as cell growth percentages relative to the controls (cells treated with dimethyl sulfoxide (DMSO) only) for each extract concentration.

### Statistical analyses

Correlation coefficients, linear regressions, and *t*-statistics of experimental data were calculated using the SPSS statistical software, version 12.0 (SPSS, Inc., Chicago, IL, USA).

## Results and Discussion

**Fig 2:**
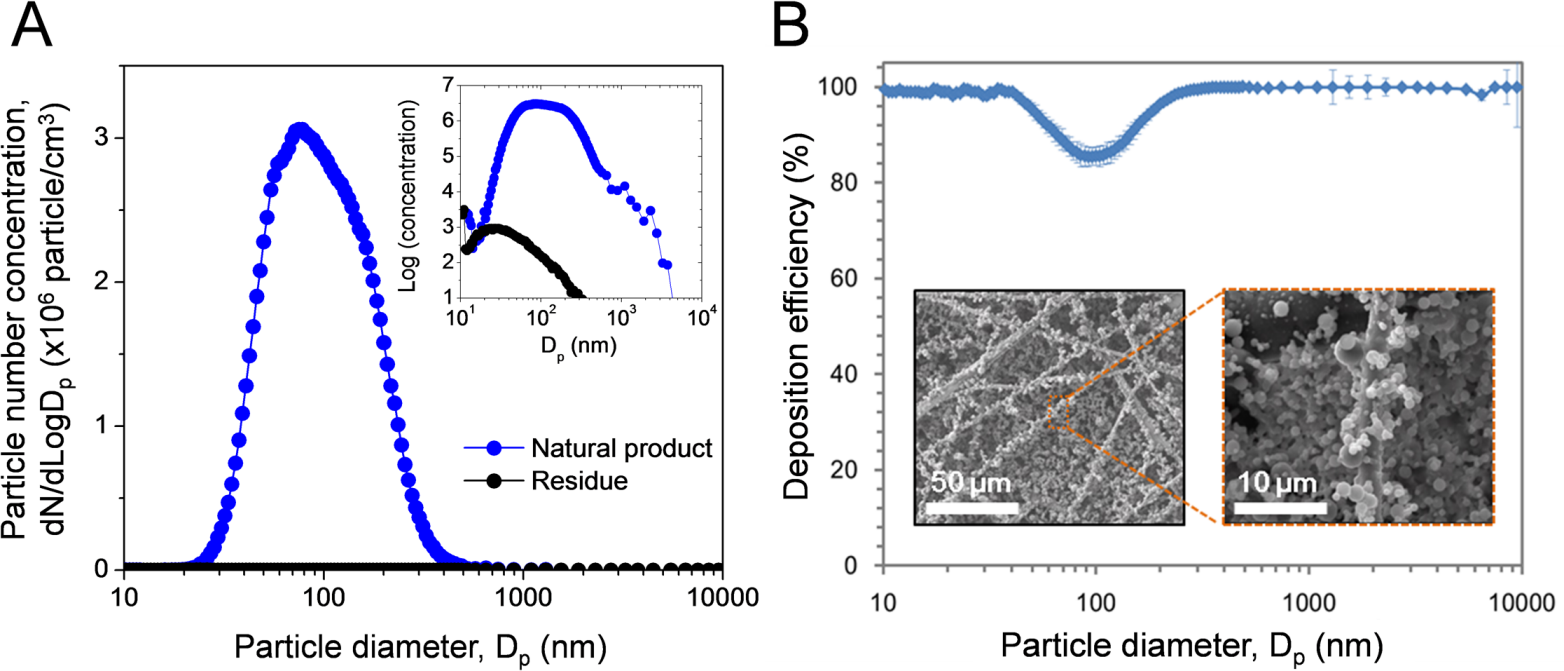
(a) Particle size distribution of natural *E*. *japonica* extract nanoparticles and residues from methanol, and (b) the fractional deposition efficiency of control filters and scanning electron micrographs (SEM) of the nanoparticles. Error bars indicate standard deviations (*n* = 3).

As shown in Fig 2 (A), nebulization and thermal drying produced natural-product nanoparticles with a wide size distribution, ranging from a few to several hundred nanometers in diameter. The distribution is best represented by a monomodal curve with a peak diameter of 75.16 nm, a geometric mean diameter (GMD) of 75.82 nm, and geometric standard deviation (GMD) of 1.217. The particles generated from methanol showed a broad size distribution, and compared to size distribution and concentration of natural-product nanoparticles, the particles generated from methanol were trivial (<3%). The deposition efficiency of these particles is shown in Fig 2 (B). More than 98% of particles with diameters less than 43 nm and greater than 277 nm were deposited onto the control filter. The deposition efficiency of particles between 43 and 277 nm was lower. The lowest deposition efficiency on the control filter was ~85.3% for particles 92 nm in diameter. The relatively low deposition efficiency observed for particles between 43 and 277 nm results from the mechanism of particle filtration. Particles with midrange diameters were too large for diffusion filtration and too small for impaction and interception mechanisms [1]. SEM micrographs show spherical and polydisperse natural-product nanoparticles on the filter fibers (Fig 2 (B)). The quantity of deposited material was determined by weighing the filters before and after particle deposition using a microbalance (Mettler MT5; Mettler-Toledo International, Inc., Seoul, Republic of Korea). The weight of the particles deposited on the filter surface ranged from ~590 to 1150 μg/cm^2^_filter_ (590, 855, and 1150 μg/cm^2^_filter_ correspond to deposition times of 3, 6, and 9 min, respectively).

**Fig 3:**
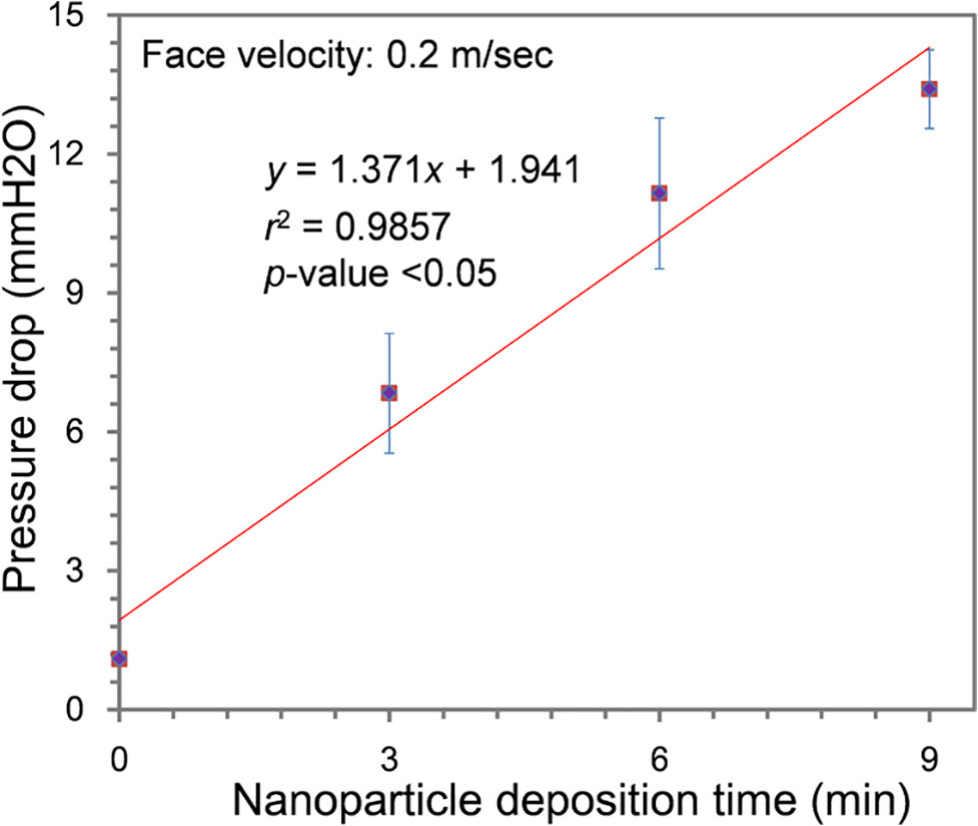
The pressure drop through the antimicrobial air filters is shown as a function of particle deposition conditions. Error bars indicate standard deviations (*n* = 3).

Fig 3 shows that pressure drop across the filter increased linearly from 1.1 to 13.4 mmH_2_O as the quantity of deposited nanoparticles increased (*y* = 1.37*x* + 1.94, *r* ^2^ = 0.9857, *p* <0.05) [60]. Filtration efficiencies were tested using *S*. *epidermidis* and *M*. *luteus* aerosols. As shown in Table 1, the test bacteria concentrations were ~278 (*S*. *epidermidis*) and ~234 particle/cm^3^ (*M*. *luteus*), respectively. The distribution of the *S*. *epidermidis* aerosol was a monomodal curve with a peak diameter of ~0.84 μm, a GMD of ~0.81 μm, and a GSD of ~1.22 (S1 Fig, Table 1). Although the shape of size distribution curve of the *M*. *luteus* aerosol was similar to that of *S*. *epidermidis* aerosol, the *M*. *luteus* particles were larger than those of *S*. *epidermidis* (S1 Fig). No statistically significant difference was observed between the filtration efficiencies of filters that had been coated for 3 and 9 min (*p* >0.05). However, because of the dissimilar size distributions of the two bacterial aerosols, filtration efficiencies of the two species were slightly different (*p* <0.05): ~97% for *S*. *epidermidis* and 95% for *M*. *luteus*. Also, note that flat panel-type filters were used in these tests to estimate the effects of natural-product nanoparticles. Real-world applications would typically employ folded filters to increase the filtration area and decrease the pressure drop.

**Table 1:** Concentrations, GSD, GMD, and peak diameters of test bacterial bioaerosols (*n* = 3).

Type of bacteria	Concentration (×10^2^ particle/cm^3^)	GSD^1^	GMD^2^ (μm)	Peak diameter (μm)
*S*. *epidermidis*	2.78 ± 0.08	1.22 ± 0.01	0.81 ± 0.01	0.84 ± 0.01
*M*. *luteus*	2.34 ± 0.03	1.42 ± 0.01	1.54 ± 0.01	1.80 ± 0.07

^1^GSD, geometric standard deviation.

^2^GMD, geometric mean diameter.

**Fig 4:**
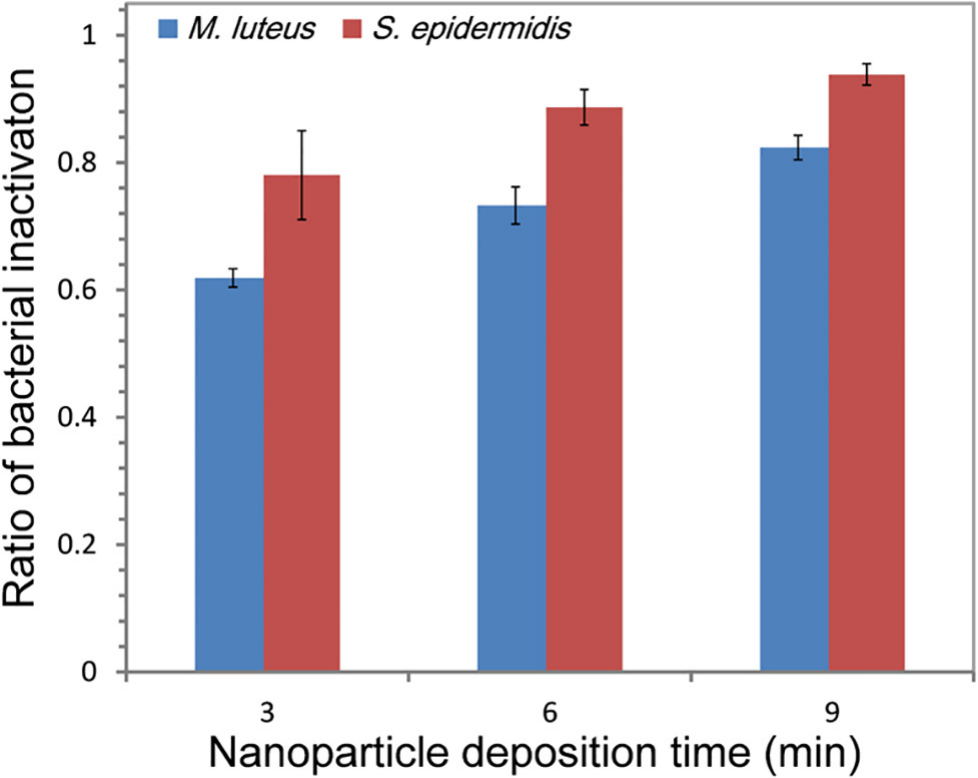
The inactivation rate of *E*. *japonica* extract nanoparticles-coated filters on bacterial aerosols. Error bars indicate standard deviations (*n* = 3).

As shown in Fig 4, the inactivation efficiencies of antimicrobial filters increased for both bacteria with increasing nanoparticle deposition time (*M*. *luteus*: *r* ^2^ = 0.9612, *S*. *epidermidis*: *r* ^2^ = 0.9959, *p* <0.05). Approximately 61, 73, and 82% of the *M*. *luteus* aerosols were inactivated on filters that had been coated for 3, 6 and 9 min, respectively, while the corresponding values were ~78, ~88, and ~94% with *S*. *epidermidis*. *M*. *luteus* was more resistant to the *E*. *japonica* extract than *S*. *epidermidis* under all conditions (*p* <0.05), with a maximum difference of ~17%. Although both *M*. *luteus* and *S*. *epidermidis* are Gram-positive bacteria, *M*. *luteus* is more suited to survive in extreme and/or nutrient-poor environments over extended periods of time [61]. Similarly, in extreme environments, *Micrococcus* strains have been shown to exhibit greater resistance to novobiocin than *Staphylococcus* strains [62].

The major chemical constituents of *E*. *japonica* extract were investigated using HPLC. The results showed that the chromatogram detected at 280 nm contains three major peaks (1–3) with relative peak areas of 16.6, 5.8, and 3.2%, respectively (S2 Fig). Positive and negative ion mode ESI/MS was used to identify these three major peaks. Peak 1 corresponded to a [M-H]^-^ ion at *m/z* 935 in negative ion mode. In positive ion mode, Peak 1 corresponded to the [M+H_2_O]^+^ ion at *m/z* 954. Fragmentation of peak 1 produced product ions at *m/z* 785 (loss of gallic acid) and *m/z* 767 (loss of gallic acid and H_2_O). Peak 1 was therefore identified as 1(ß)-*O*-galloyl pedunculagin. The presence of ions at *m/z* 301 or 284 in the mass spectra of peaks 2 and 3 indicated that these compounds were quercetin and kaempferol derivatives, respectively. Peak 2 yielded a [M-H]^-^ ion at *m/z* 477 and another characteristic fragment at *m/z* 301, corresponding to the loss of a glucuronide moiety. Peak 2 was therefore assigned to quercetin-3-*O*-glucuronide. Peak 3 yielded a [M-H]^-^ ion at *m/z* 447 (corresponding to an aglycone) and a major fragment ion at *m/z* 284 due to the loss of a hexose unit, and was identified as kaempferol-3-*O*-glucoside. These results were confirmed by comparisons with MS data in previous reports [63–65]. Previous studies showed that flavonoids quercetin-3-*O*-glucuronide and kaempferol-3-*O*-glucoside exhibited antibacterial activity by reducing the fluidity of the inner and outer layers of bacterial membranes, inhibiting DNA and RNA synthesis, and deterring energy metabolism [66]. Quercetin-3-*O*-glucuronide showed stronger antibacterial activity and a lower acute toxicity than kaempferol-3-*O*-glucoside [67]. The antibiotic activities of 1(ß)-*O*-galloyl pedunculagin are not well known. Thus, quercetin-3-*O*-glucuronide and kaempferol-3-*O*-glucoside likely play important roles in determining the antimicrobial efficacy of *E*. *japonica*.

**Fig 5:**
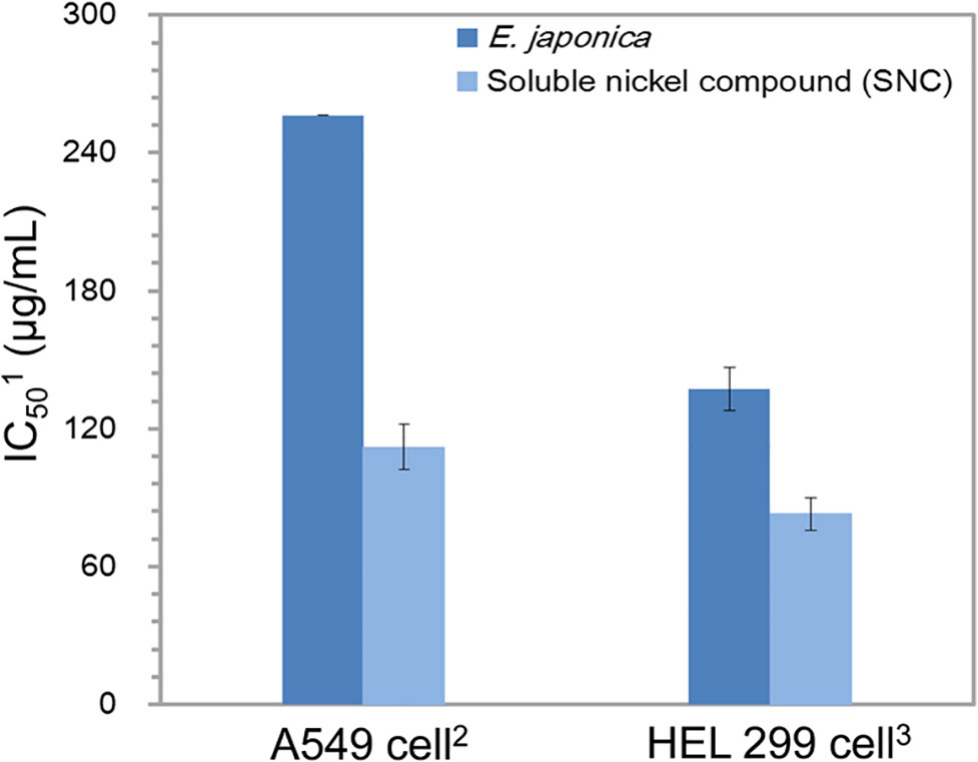
The inhibitory effects of *E*. *japonica* and a soluble nickel compound (SNC) on A549 cancer and HEL 299 cells. Error bars indicate standard deviations (*n* = 10) ^1^Half maximal inhibitory concentration, ^2^A549 human lung adenocarcinoma cancer cells, ^3^HEL 299 human lung fibroblast cells.

To assess the toxicity of *E*. *japonica*, *in vitro* cytotoxicity tests were conducted using EZ-Cytox cell viability assay kits, the results of which were compared with those of SNCs, which are known to produce genotoxic effects in cells. The SNC used in this study is classified as a human carcinogen by the U.S. National Toxicology Program (NTP) and Beraterkreis Toxikologie in Germany [68] although it is a weaker carcinogen than other, insoluble nickel compounds. Fig 5 shows the concentration of *E*. *japonica* and SNC required to attain 50% inhibition of A549 cancer cells and HEL 299 cells. For both cell types, the inhibitory concentration of *E*. *japonica* was statistically confirmed to be higher than that of the SNC following 48-h exposures (*p* <0.05). The required *E*. *japonica* concentrations were 137 and 256 μg/mL, >57% higher than those of the SNC.

**Fig 6:**
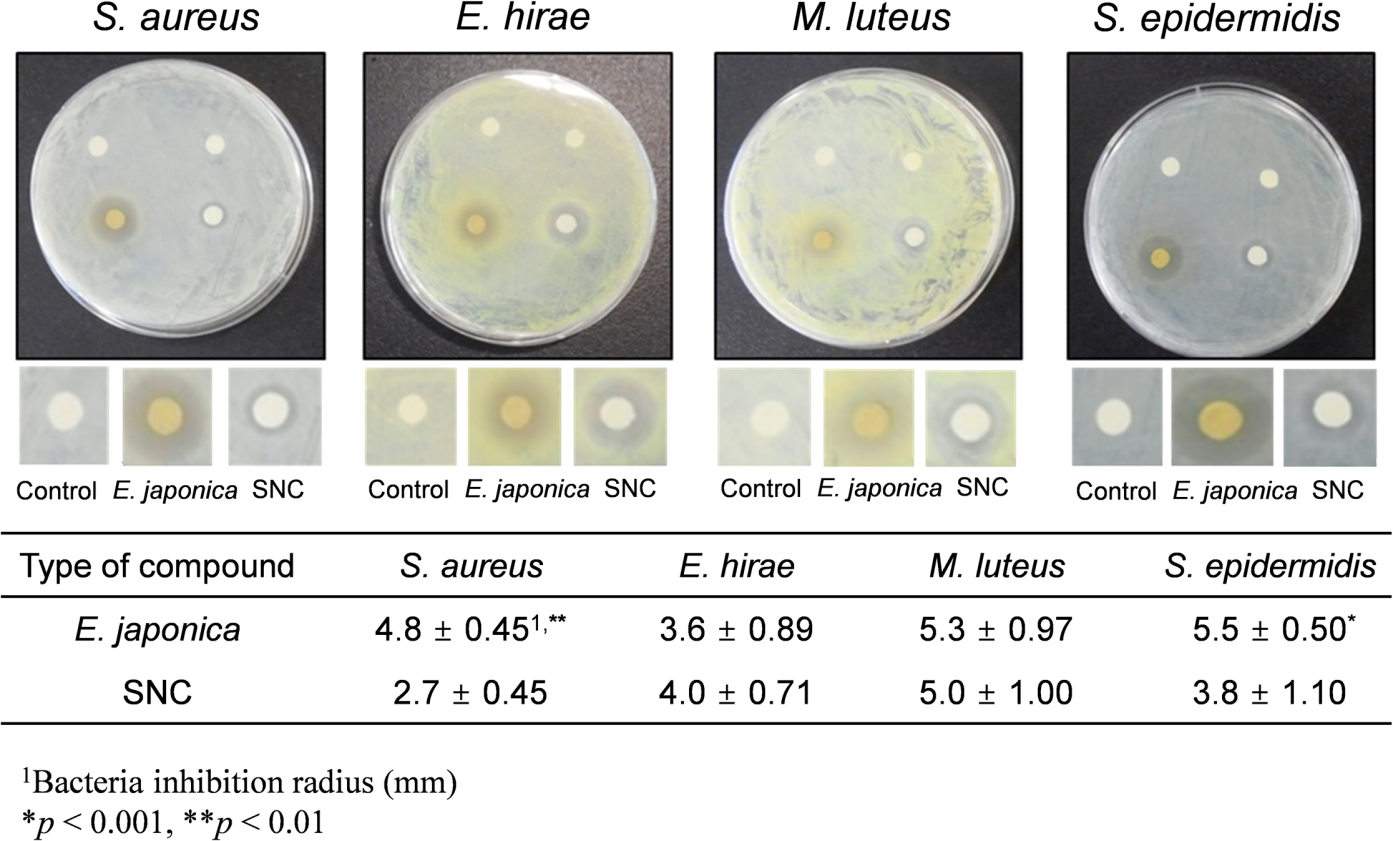
A comparison of the antimicrobial activity of *E*. *japonica* and SNC using the disk diffusion method (*n* = 5).

In addition, the antimicrobial activities of *E*. *japonica* and the SNC were casually evaluated using the disk diffusion method. Approximately 50 mg of *E*. *japonica* extract and SNC powders were dissolved in 1 mL of DMSO and distilled water, respectively, and 10 μL of each suspension was used to soak Whatman filter papers (11-mm diameter; GE Healthcare Life Sciences, Pittsburgh, PA, USA). Fig 6 shows the results against *S*. *aureus*, *Enterococcus hirae*, *M*. *luteus*, and *S*. *epidermidis*. The antimicrobial activities of *E*. *japonica* were similar to those of the SNC against *E*. *hirae* and *M*. *luteus* (*p* >0.05), while it was considerably more effective against *S*. *aureus* and *S*. *epidermidis* with inhibition zones extending to more than twice the radius of those of the SNC (*p* <0.01) The results in Figs 5 and 6 confirm that *E*. *japonica* is less toxic and exhibits stronger antimicrobial activity on some bacteria than the SNC.

## Conclusion

Filters coated with natural *E*. *japonica* extract nanoparticles are effective in inactivating bioaerosols. The pressure drop and antimicrobial activity of nanoparticle-coated filters increased with increasing nanoparticle deposition time. HPLC and ESI/MS analyses showed that the major chemical compounds in the *E*. *japonica* extract were 1(ß)-*O*-galloyl pedunculagin, quercetin-3-*O*-glucuronide, and kaempferol-3-*O*-glucoside. The latter two compounds likely play important roles in the inactivation of bacterial aerosols. *In vitro* cytotoxicity and disk diffusion tests showed that *E*. *japonica* nanoparticles were less toxic and had stronger antimicrobial activity on some bacterial strains than the SNC, which is classified as a human carcinogen. Note that the amount of nanoparticles deposited on a given filter must be optimized for the type of bacterial aerosol. The antimicrobial performance of the *E*. *japonica* nanoparticle-coated filters depended both on the amount of nanoparticles deposited and the nature of the airborne bacteria. Additionally, previous studies showed that major chemical components in a natural product were naturally degraded over time at room temperature [69] and antimicrobial activity and morphologies of natural-product nanoparticles were affected by a variety of environmental factors such as humidity and temperature [40,70]. Thus, the long-term stability and effects of humidity and thermal energy on *E*. *japonica* nanoparticles need to be evaluated to estimate the efficiency of nanoparticle-coated filters for real-world applications. Although the *in vitro* tests showed that *E*. *japonica* is less toxic than the carcinogen, it does not mean that the natural product is harmless to human health. Thus, additional studies such as *in vivo* experiments are required. In a biological setting, many experimental values should be considered. Thus, field experiments are required to confirm the applicability of the natural-product nanoparticle-coated filter in a real environment. We are considering a field experiment of our filters in a future study. This study provides valuable information for the development of an environmentally friendly bioaerosol control system that is suitable for use in indoor environments.

## Supporting Information

S1 Fig The size distribution of test bacterial bioaerosols. (TIF)

S2 Fig (a) HPLC chromatogram of *E*. *japonica* extract detected at 280 nm and (b) mass spectra of major compounds in the *E. japonica* extract. (TIF)
