# Supplementary figures and images for: Antimicrobial Air Filters Using Natural Euscaphis japonica Nanoparticles
Source: PLoS One. 2015 May 14;10(5):e0126481. doi: 10.1371/journal.pone.0126481 (PMC4431859; doi:10.1371/journal.pone.0126481)

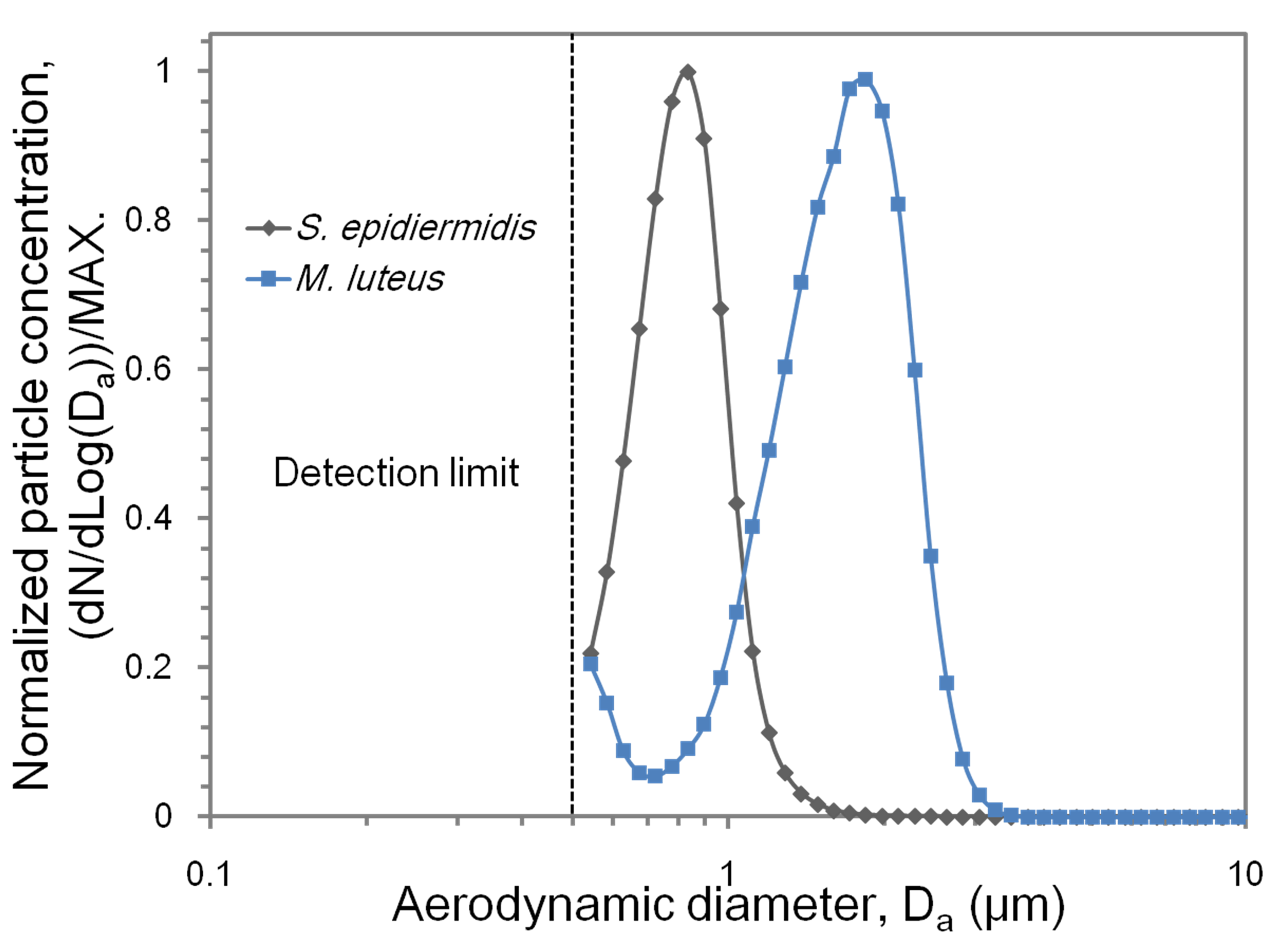

Supplement: S1 Fig — (TIF) [file pone.0126481.s001.tif]

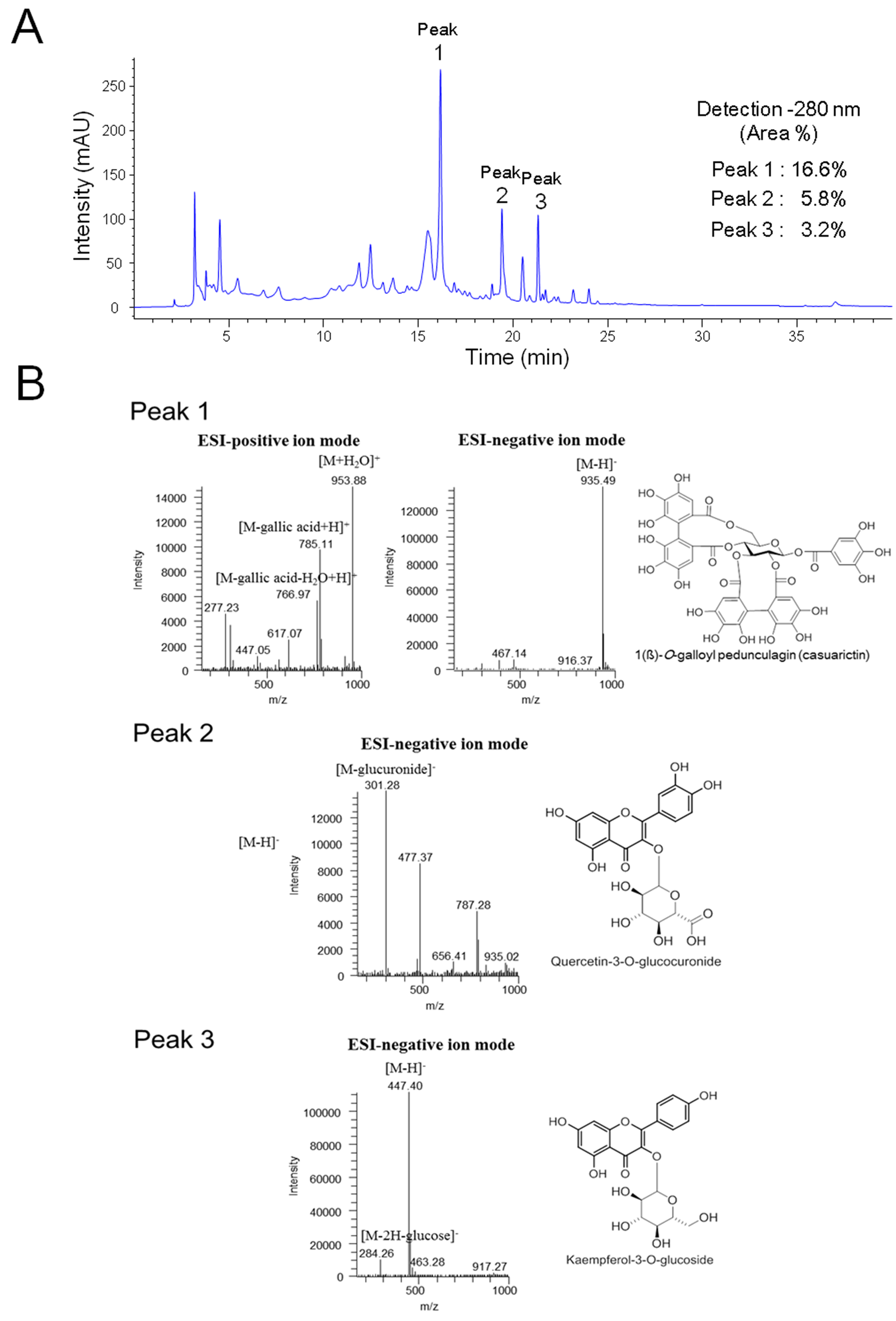

Supplement: S2 Fig — (TIF) [file pone.0126481.s002.tif]
